# The Emergence of Dirac points in Photonic Crystals with Mirror Symmetry

**DOI:** 10.1038/srep08186

**Published:** 2015-02-02

**Authors:** Wen-Yu He, C. T. Chan

**Affiliations:** 1 Department of Physics and Institute for Advanced Study, the Hong Kong University of Science and Technology, Clear Water Bay, Hong Kong, China

## Abstract

We show that Dirac points can emerge in photonic crystals possessing mirror symmetry when band gap closes. The mechanism of generating Dirac points is discussed in a two-dimensional photonic square lattice, in which four Dirac points split out naturally after the touching of two bands with different parity. The emergence of such nodal points, characterized by vortex structure in momentum space, is attributed to the unavoidable band crossing protected by mirror symmetry. The Dirac nodes can be unbuckled through breaking the mirror symmetry and a photonic analog of Chern insulator can be achieved through time reversal symmetry breaking. Breaking time reversal symmetry can lead to unidirectional helical edge states and breaking mirror symmetry can reduce the band gap to amplify the finite size effect, providing ways to engineer helical edge states.

Topologically characterized gapless points induce many novel phenomena in both electronic and photonic systems, from robust edge states to back-scattering immune transport^1^,^2^,^3^,^4^,^5^,^6^,^7^,^8^,^9^,^10^,^11^. At the band touching point, vortex structure for Dirac nodal points or monopole for Weyl nodes appear in the momentum space as the topological feature. As these remarkable properties are mainly attributed to the topological nature of these nodal points, the mechanism of the creation, moving and vanishing of them, attracts significant research attention both theoretically and experimentally^6^,^7^,^12^,^13^,^14^,^15^. In previous studies, the creation and manipulation of Dirac points is typically involved with symmetry operations. For Dirac nodes in honeycomb or square lattices, lattice anisotropy is necessary to induce the topological transition^6^,^13^,^14^,^15^, while for Weyl nodes in double gyroid photonic crystals, either parity or time reversal symmetry breaking is required^7^. However, interesting band degeneracy phenomenon also occurs through the variation of parameters unrelated to symmetry, as the exotic topological semimetal phase in Ref. 16 and the photonic Dirac cone at Brillouin zone center in Ref. 17. Such results motivate us to explore the possibility to produce and manipulate Dirac points in an unconventional way.

In this paper, we show theoretically that Dirac points can potentially emerge in all photonic crystals with mirror symmetry during the closing of a photonic band gap. When the mirror symmetry is present, the eigenmodes along the high symmetry line that are invariant under the mirror operator can be classified by the mirror symmetry representation. Once two bands with different parity are tuned to approach each other, band crossing is unavoidable after their touching. Such unavoidable crossing induced band degeneracy is stable and cannot be destroyed unless the mirror symmetry is broken. This mirror symmetry can generate Dirac points for 2D lattice, and is responsible for the edge states of the 3D topological crystalline insulator^18^,^19^. We investigate in detail the property of such band degeneracy in a photonic square lattice comprising dielectric cylinders. The photonic square lattice possesses two equivalent mirror planes, generating two pairs of Dirac points as expected. With k·p perturbation and symmetry analysis using group theory, we obtain the effective Hamiltonian near the degeneracy and present the evolution of subbands to show the mechanism of generating Dirac points. Subsequently the effect of mirror symmetry breaking is studied through replacing dielectric cylinders, which have mirror symmetry in any direction, with artificial structures, which only have mirror symmetry along its principal axis. We found that fixing its principal axis parallel to one of the mirror planes would lift one pair of Dirac points and preserve the other, while a general orientation would break both Dirac points, as is consistent with its mirror symmetry protection. The topological feature of the Dirac points is manifested by considering the magneto-optical effect. A photonic Chern “insulator” with two helical edge states is realized due to the breaking of time reversal symmetry (here photonic “insulator” means the propagation of light at a certain frequency range is forbidden by an absolute photonic band gap). Furthermore, introducing such special artificial structure to this photonic Chern “insulator”, the finite size effect^20^ is found to be amplified, which suppresses one helical edge state while does not influence the other at specific frequency.

## Results

### Dirac points and mirror symmetry

Consider a photonic crystal possessing mirror symmetry, such mirror reflection invariance gives
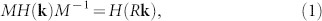
where *M* is the mirror operator and *R* is the 2 × 2 mirror reflection matrix defining the mirror reflection in a 2D plane. In the Brillouin zone, a mirror reflection invariant line satisfies the condition *R***k** = **k** + **G** (where **G** is reciprocal lattice vector). Along the mirror reflection invariant line, the eigenfunction of the Hamiltonian is also an irreducible representation of the mirror operator, and thus all bands along that direction can be labeled by {A, B}, which correspond to the representation with even and odd parity respectively under the mirror operator. If two bands with different parity get close to each other, the effective Hamiltonian can be approximated on the basis of A and B. On this basis, the matrix representation of *M* is *σ_z_*. Along the mirror reflection invariant line, the symmetry constraint upon the effective Hamiltonian makes its off diagonal elements become zero and only leaves diagonal terms. Such diagonal terms control the dispersion of the two bands independently, and the two bands can have unavoidable linear crossing once after their touching, as is shown in Fig. S1. Near the crossing point, expanding the effective Hamiltonian to the first order subject to symmetry constraints, we can generate the effective Hamiltonian as the following
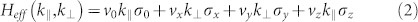
where *k*_∥_ and *k*_⊥_ represent the vector component parallel and perpendicular to the mirror reflection invariant line respectively. The detailed illustration about the symmetry constraint is given in the Supplemental Information. The crossing point has a linear dispersion in all directions and acts as a source (sink) of Berry curvature in momentum space. When the dielectric constant of cylinders is very high, a photonic crystal has band gaps separating isolated bands. As we progressively reduce the dielectric constant, the photonic bands will close as some bands separated by the band gaps will approach and touch each other. In this process, if mirror symmetry is present, touching between photonic bands in the mirror reflection invariant line with different parity will make Dirac points split out naturally. This intrinsic connection between band gap closing and emergence of Dirac points provides the possibility for creating Dirac points in photonic crystals with mirror symmetry.

### Emergence of Dirac points

**Figure 1:**
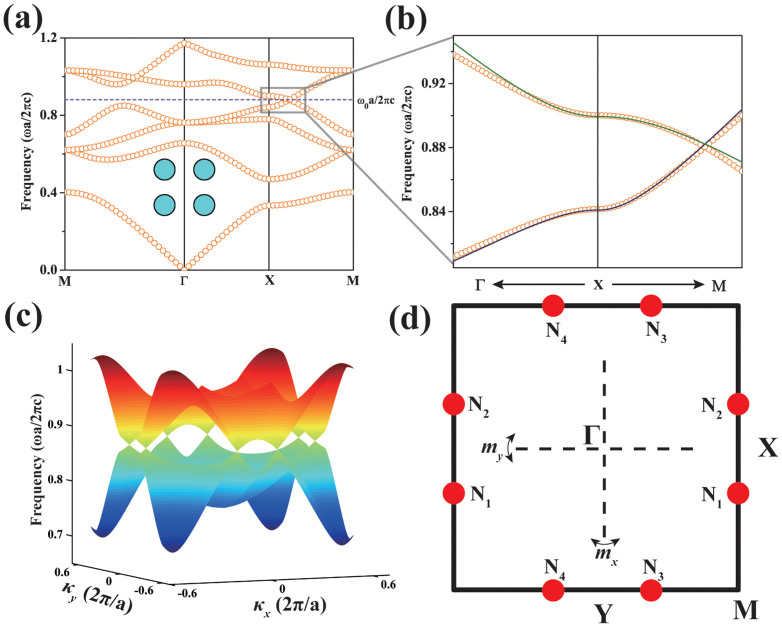
Band structure of a 2D photonic crystal with a square lattice. The photonic crystal is composed of dielectric cylinders as shown in the inset in panel (a) with ε = 5.4 and radius r = 0.2a embedded in air. Here, a is the lattice constant. (a) The band structure along high symmetry lines. Gapless Dirac points with linear dispersions at frequency ω_0_ emerge along XM. (b) Enlarged view near the Dirac point, with the solid lines showing k·p perturbation result. (c) Three dimensional dispersion surface containing four Dirac points in the first Brillouin zone. (d) The red dots mark the positions of Dirac points {N_1_, N_2_, N_3_, N_4_} in the first Brillouin zone. The two perpendicular dot lines represent the mirror operation *m_x_* and *m_y_*.

We consider a two-dimensional photonic crystal system in a square lattice consisting of dielectric cylinders. We perform plane wave expansion^21^ to calculate the band structure for the transverse magnetic (TM) polarization with the electric field along the rod axis. Here we denote *a* as the lattice constant, and relative permittivity and radius of the cylinders are ε = 5.4, r = 0.2*a* respectively. Along the XM direction, the fourth and fifth bands cross linearly at frequency ω_0_, as is shown in Fig. 1(a), suggesting that isolated band degeneracy occurs in the momentum space. Further calculation of three-dimensional band dispersion demonstrates that band touching with linear dispersion arises around X and Y as shown in Fig. 1(c) and (d), giving rise to four gapless points in the first Brillouin zone. Emergence of Dirac points can also appear in other lattices as long as there is a mirror symmetry. For example, six Dirac points can also emerge in a photonic crystal with triangular lattice as can be seen in Fig. S2 in the Supplementary Information. This phenomenon is stable and immune to small variation in radius and permittivity of rods, which can only shift degenerate points in frequency and momentum space but cannot remove them. The band degeneracy vanishes once a pair of degeneracy points meet at X (Y), where they split out, and pairwise annihilation occurs. Such characteristics of the degeneracy points resemble the behaviors of Weyl fermions^22^,^23^, indicating the topological nature of the Dirac points.

### k·p perturbation and symmetry protection

The Maxwell equation for TM wave in two dimensional photonic crystals can be converted to an eigen problem. We can express the Bloch wave function *ψ_n_***_k_** as a linear combination of *ψ_n_***_k0_** with eigenfrequency *ω_n_*_0_, and establish an effective Hamiltonian with matrix element 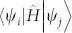 to describe the photonic system^24^. For the square lattice, all symmetry operations in the C_2V_ group will keep the X (Y) point invariant, suggesting that the symmetry type of eigenfunction at X (Y) belongs to {B_2_, A_1_, B_1_, A_2_}, the irreducible representations of C_2V_^25^. For this reason we adopt 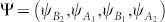, which corresponds to the eigenfunction for the third, fourth, fifth, and sixth band respectively, as the basis wave function to do *k*·*p* perturbation near the X point (Here the Bloch wave functions are obtained through COMSOL Multiphysics, a commercial package based on the finite-element method). Then a 4 × 4 effective Hamiltonian can be deduced as follows
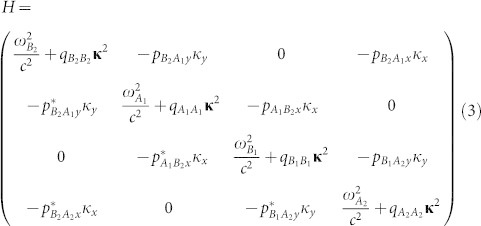
Here **κ** **=** **k−k_0_**, c is the speed of light, ω*_i_* (*i*∈{B_2_, A_1_, B_1_, A_2_}) is the eigen frequency at X for corresponding band and the coefficient is 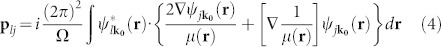
and
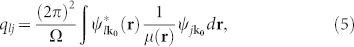
which can be obtained by numerical integration of the eigen wave function through the unit cell. The Ω here is the area of the unit cell to normalize the wave function. Results from this effective Hamiltonian generate eigen spectrum that agree well with plane wave calculation as can be seen from Fig. 1 (b), and obtain the weight of each representation simultaneously. From group theory, this effective Hamiltonian should keep invariant under any symmetry operation of C_2V_, which means that nonzero Hamiltonian matrix element only exists when the direct product of the irreducible representations of *ψ_i_*, 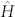, and *ψ_j_* contains A_1_, the full symmetry representation^26^. It can be confirmed that this effective Hamiltonian is consistent with the requirement by group theory, indicating the emergence of such Dirac points is intrinsically decided by the crystal symmetry.

**Figure 2:**
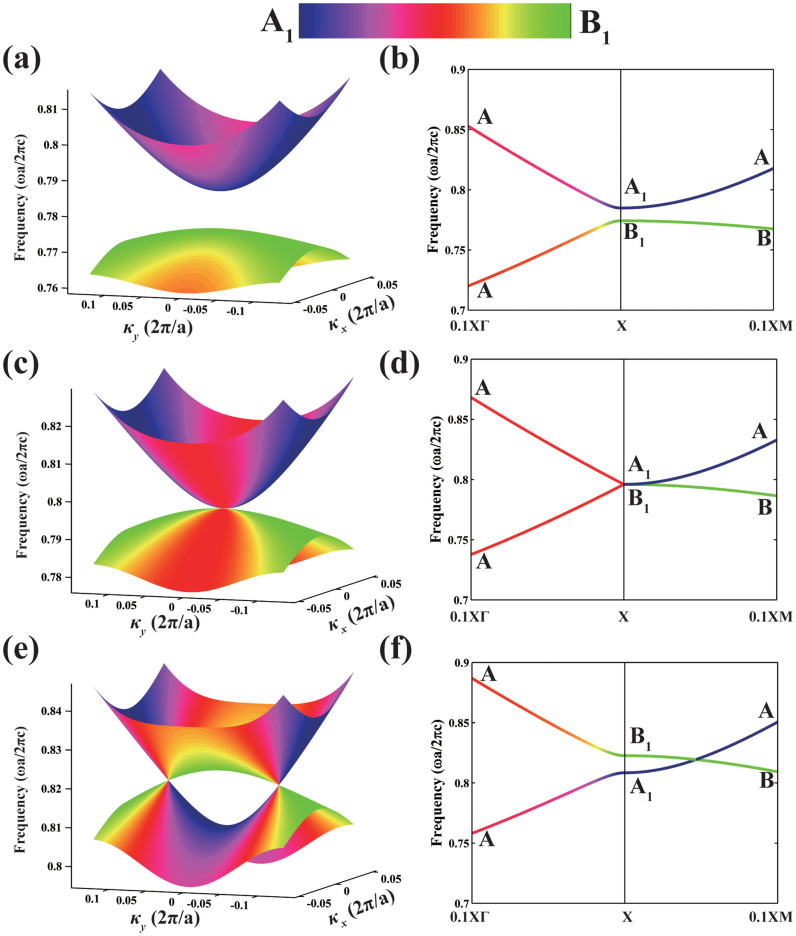
Evolution of A1 and B1 subbands with variation of cylinders' relative dielectric constant. Dirac points emerge as the photonic band gap closes upon a reduction of the dielectric constant. The relative permittivity and radii adopted are {ε = 8.8, r = 0.2a} for (a) and (b), {ε = 8, r = 0.2a} for (c) and (d), {ε = 7.2, r = 0.2a} for (e) and (f). The colored shading indicates the symmetry type of the band at the corresponding point in Brillouin zone. A and B are irreducible representation of C_1h_ group with even and odd parity respectively. We note that the bands repel along ΓX after the A_1_ and B_1_ touch and change order when the band gap closes, but the bands along XM have an unavoidable crossing giving rise to Dirac points.

From the effective Hamiltonian, we see that it is block diagonalized along ΓX and XM direction. For κ*_x_* = 0 along XM, A_1_ state only couples with B_2_ state while B_1_ state only couples with A_2_ state, reducing A_1_ state and B_1_ state to A and B type respectively (here A and B are irreducible representations of mirror operator *m_x_*). As A and B bands approach each other when the photonic band gap closes due to the reduction of ε in the cylinders, band crossing would unavoidably occur along XM. Such unavoidably crossing is intrinsically protected by the mirror symmetry of *m_x_*, and guarantees the emergence of degeneracy at N_1_ and N_2_. However, for κ*_y_* = 0 along ΓX, A_1_ and B_1_ state are both reduced to A type. Thus in the process of band gap closing, level repulsion and inversion between the two A type bands takes place once they touch. Such band inversion and repulsion induces directional band gap along ΓX, acting as the role of partial gap to isolate the two band crossing points and making a pair of Dirac points N_1_ and N_2_ split out, as is shown in Fig. 2. We see here that the Dirac points N_1_ and N_2_ are stable and generic once they emerge, as long as the *m_x_* mirror symmetry protected unavoidable crossing maintains. Such analysis is also suitable for Dirac points N_3_ and N_4_ near Y, which are protected by *m_y_* mirror symmetry (the calculation details for the evolution of A_1_ and B_1_ states is in the Supplemental Information).

Since the Dirac points are formed by the linear crossing of the fourth and fifth bands, far away from the third and sixth bands in frequency, bands near Dirac degeneracy are mainly formed the mixture of A_1_ and B_1_ states, while fraction of A_2_ and B_2_ is tiny small. It is therefore appropriate to apply second order perturbation to integrate out the distant frequency degrees of freedom and reduce the effective Hamiltonian to a two band model as
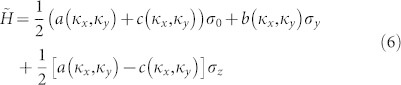
where *σ_y_* and *σ_z_* are two Pauli matrices and *I* is the 2 × 2 identity matrix. This effective Hamiltonian is approximated by eigen states at X (Y) point, and it can be reduced to Eq. (2) through Taylor expansion around the degenerated Dirac points. Using the coefficients of Pauli matrices to define a planar vector **h** = (*h_x_*,*h_y_*), with *h_x_* = *b*(*κ_x_*, *κ_y_*) and *h_y_* = 1/2[*a*(*κ_x_*, *κ_y_*)-*c*(*κ_x_*, *κ_y_*)], we can interpret the Dirac points as a topological vortex, with topological index (the winding number) defined as^16^
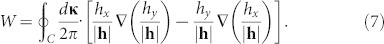
The 2D planar vector **h** around each time reversal related Dirac point indeed has vortex structure with nontrivial winding number 1 and −1, while the winding number vanishes once both time reversal partners are included, as is shown in Fig. S3. This is distinct from the Dirac cones splitting out of quadratic degeneracy by symmetry breaking operation in Ref. 27, where the winding number around each cone is 1 and around the both is 2 instead. This vortex structure in momentum space manifests the topological feature of the nodal points.

**Figure 3:**
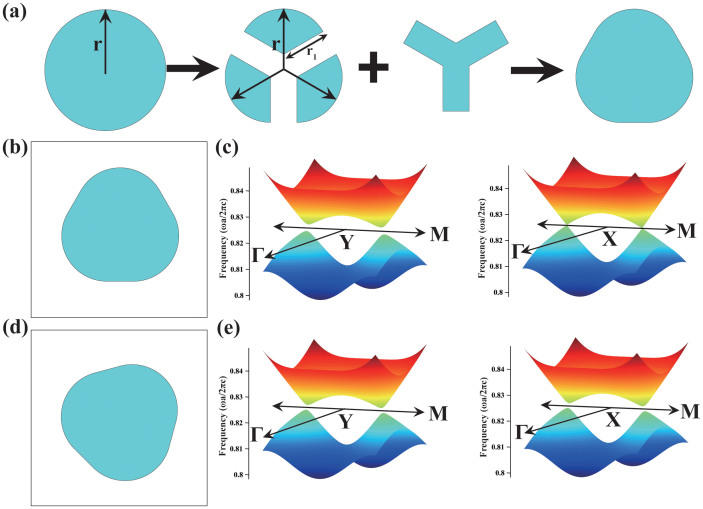
The effect of mirror symmetry breaking on the band structure. (a) Design of an artificial structure with a smooth transition from cylinder to triangular shape. First, the cylinder is narrowed and trisected. Then the empty area between the three sectors is filled with the same material allowing for a smooth profile of the whole structure. For (b) the special artificial structure of {ε = 7.2, r_1_ = 0.18a, r = 0.2a} in the unit cell aligns its principal axis parallel to a lattice unit vector while it is rotated by 45° for (d). (c) The band structure near lifted degenerate points around Y and maintaining Dirac points around X. (e) The band structure near lifted degenerate points around both Y and X.

We now consider symmetry breaking effect on these Dirac points. An artificial structure is shown in Fig. 3(a), where the ratio between r_1_ and r can be used to describe the degree of its deviation from cylinder (it is still a cylinder when r_1_ = r). Here we found that the principal axis of the artificial structure orientating parallel to the mirror plane of *m_x_* would lift one pair of Dirac nodes N_3_ and N_4_ while keep the other pair N_1_ and N_2_, as is shown in Fig. 3(b) and (c), while a general orientation of the principal axis would break both, as is shown in Fig. 3(d) and (e). This is due to the fact that the former case maintains mirror symmetry of *m_x_*, but breaks that of *m_y_*, while the latter case both the mirror symmetry of *m_x_* and *m_y_* is broken. Consequently the breaking of mirror symmetry induces hybridization between A and B type bands so that crossing is avoided and Dirac nodes are unbuckled. This is consistent with the previous analysis that the stability of Dirac points vanishes as the mirror symmetry protected unavoidable crossing fails. Here, the coexistence of buckled and unbuckled Dirac nodes is achieved in this photonic system and the width of gap for unbuckled ones is also found to be tunable through varying the degree of deformation. This phenomenon gives us the possibility to manipulate the Dirac points through controlling the mirror symmetry of such special artificial structure.

### Photonic Chern “insulator”

**Figure 4:**
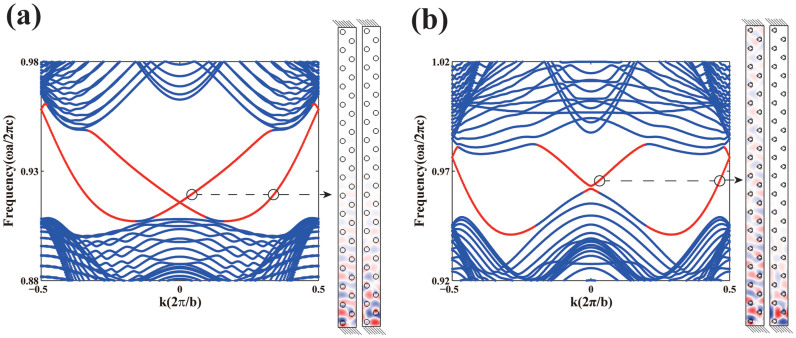
Topological edge states induced by magneto-optical effect. The projected band structure of the photonic crystal ribbon containing 33 layers with PEC boundary (the shaded area at both ends means the PEC boundary) at the end is shown for structural parameters {r_1_ = 0.2a, r = 0.2a} for (a) and {r_1_ = 0.1a, r = 0.2a} for (b). The inset on the right shows the electric field distribution of the two edge modes marked by circle arranged in the same order. In (b), the mirror symmetry breaking along the helical propagating direction strengthens the finite size effect and opens a gap in edge mode spectrum. In both cases ε = 5.4, μ = μ_0_ = 1, and μ_κ_ = 0.5 are taken. Here the periodicity of the ribbon is denoted by 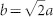.

For photonic crystals with such Dirac nodes, nodes unbuckling by time reversal symmetry breaking will introduce nontrivial Chern numbers. We use a magnetic permeability tensor with imaginary off-diagonal components to represent the magneto-optical effect and break the time reversal symmetry^28^. It has the following form
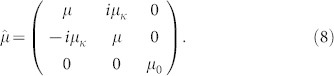
Introducing magneto-optical effect breaks parity^27^, unbuckles all four Dirac nodes, and nonzero Chern number emerges. Since the integration of Berry curvature near each node contributes to a Chern number of ± 1/2^29^, the unbuckled four Dirac nodes in our case give rise to a photonic Chern “insulator” with Chern number |C| = 2. Consistent with the bulk-edge correspondence^30^,^31^, two different edge states emerge at the boundary of this photonic system, as is shown in Fig. 4 (a).

A very recent paper^32^ shows that multimode one-way waveguide of large chern number becomes realizable with this mechanism. Since the number of equivalent mirror planes in photonic crystal potentially determines how many pairs of Dirac points can be created, breaking time reversal symmetry by magneto optical effect straightforward combines these with Chern number. It indicates that for the triangular lattice (see Fig. S2), photonic Chern “insulator” with three helical edge state is expected.

### Enhancement of finite size effect

The helical edge states along the boundary of photonic Chern “insulator” has unidirectional propagation^2^,^9^,^33^, and the strength of the modes decays exponentially in the direction perpendicular to the boundary. Owing to the finite size of the sample, the edge mode can leak to the other side and couple with its counterpart to induce a gap in the edge spectrum. This is a typical finite size effect^20^. Interestingly, we find such finite size effect is amplified by mirror symmetry breaking along the propagating direction (here we replace dielectric cylinders with a special artificial structure and align its principal axis along propagating direction). When we compare Fig. 4(b) with Fig. 4(a), we see that the band gap size is reduced when the mirror symmetry is broken. The reduced band gap leads to a stronger coupling of the edge mode on either side of the finite-sized sample, opening a minigap in the edge mode spectrum. As is shown in Fig. 4(b), there exists a gap in the edge modes spectrum at k = 0 and one edge mode is observed to leak to the other side with decay length comparable to the width of the ribbon. Moreover, only widening the photonic supercell and keeping all other condition invariant would suppress the gap in the edge spectrum drastically and reduces the leakage of the corresponding edge mode dramatically, as is shown in Fig. S5 in the Supplementary Information, confirming that it is finite-size effect. Since the edge spectrum of the two edge modes crosses at different frequencies, such enhanced finite size effect only affect one edge mode at degenerate frequency and does not influence the other. It seems that based on this mechanism, the two helical edge states become controllable and a frequency dependent helical wave filter is feasible.

## Conclusion

In summary, we have shown that Dirac points can potentially split out in photonic crystals with mirror symmetry during the closing of photonic gaps by the touching of two bands with different parity. The full property of Dirac points, including the emergence and the response to symmetry breaking effect, is then investigated in a photonic square lattice. Such an effect can enable the design of photonic Chern “insulator” with large Chern number, manipulation of multi-helical edge states, photonic valleytronics, and the development of novel wave functional devices.

## Supplementary Material

Supplementary Information The Emergence of Dirac Points in Photonic Crystal with Mirror Symmetry
