# Supplementary material for: The Emergence of Dirac points in Photonic Crystals with Mirror Symmetry
Source: Sci Rep. 2015 Feb 2;5:8186. doi: 10.1038/srep08186 (PMC4650825; doi:10.1038/srep08186)
Supplement: Supplementary Information — The Emergence of Dirac Points in Photonic Crystal with Mirror Symmetry [file srep08186-s1.pdf]

The Emergence of Dirac Points in Photonic Crystal with Mirror Symmetry

Wen-Yu He and C. T. Chan*

Department of Physics and Institute for Advanced Study, Hong Kong University of Science and Technology, Clear Water Bay, Kowloon, Hong Kong, China

Supplementary Information

I. Derivation of the effective Hamiltonian using symmetry constraints

In general, the 2×2 effective Hamiltonian is given by

$$H_{eff}\left(k_{\parallel},k_{\perp}\right)=a\left(k_{\parallel},k_{\perp}\right)\sigma_0+b\left(k_{\parallel},k_{\perp}\right)\sigma_x+c\left(k_{\parallel},k_{\perp}\right)\sigma_y+d\left(k_{\parallel},k_{\perp}\right)\sigma_z$$

Under the mirror operator, the effective Hamiltonian must behave like the following

$$MH_{eff}\left(k_{\parallel},k_{\perp}\right)M^{-1}=H_{eff}\left(k_{\parallel},-k_{\perp}\right)$$

On the basis of eigenfunction with different parity, the matrix representation of mirror operator is σ_z. Thus along the mirror reflection invariant line, $\sigma_z H_{eff}\left(k_{\parallel}\right)\sigma_z^{-1}=H_{eff}\left(k_{\parallel}\right)$ gives

$$d_0\left(k_{\parallel}\right)\sigma_0+d_x\left(k_{\parallel}\right)\sigma_x+d_y\left(k_{\parallel}\right)\sigma_y+d_z\left(k_{\parallel}\right)\sigma_z=d_0\left(k_{\parallel}\right)\sigma_0-d_x\left(k_{\parallel}\right)\sigma_x-d_y\left(k_{\parallel}\right)\sigma_y+d_z\left(k_{\parallel}\right)\sigma_z$$

It makes σ_x and σ_y, the off diagonal terms vanish. Considering the perpendicular component, it gives that

$$d_0\left(k_{\parallel},k_{\perp}\right)\sigma_0+d_x\left(k_{\parallel},k_{\perp}\right)\sigma_x+d_y\left(k_{\parallel},k_{\perp}\right)\sigma_y+d_z\left(k_{\parallel},k_{\perp}\right)\sigma_z=d_0\left(k_{\parallel},-k_{\perp}\right)\sigma_0-d_x\left(k_{\parallel},-k_{\perp}\right)\sigma_x-d_y\left(k_{\parallel},-k_{\perp}\right)\sigma_y+d_z\left(k_{\parallel},-k_{\perp}\right)\sigma_z$$

Expanding them near the crossing point to the first order and following the restriction that $b(k_{\parallel}, k_{\perp})$ and $c(k_{\parallel}, k_{\perp})$ are both odd in $k_{\perp}$, we obtain

$$d_0\left(k_{\parallel},k_{\perp}\right)=v_0k_{\parallel}$$

$$d_x\left(k_{\parallel},k_{\perp}\right)=v_xk_{\perp}$$

$$d_y\left(k_{\parallel},k_{\perp}\right)=v_yk_{\perp}$$

$$d_z\left(k_{\parallel},k_{\perp}\right)=v_zk_{\parallel}$$

The eigen value of this effective Hamiltonian reads

$$\Delta\omega=v_0k_{\parallel}\pm\sqrt{v_z^2k_{\parallel}^2+\left(v_x^2+v_y^2\right)k_{\perp}^2}$$

II. Symmetry analysis with group theory in the **k·p** perturbation

The effective Hamiltonian matrix is mainly decided by the coefficient (p_{ljx}, p_{ljy}) and q_{lj} [S2]. The magnetic permeability of the cylinders is assumed to be one and then the expression of $\mathbf{p}_{lj}$ and q_{lj} can be simplified as

$$p_{ljx} = i\frac{(2\pi)^2}{\Omega}\int \psi^*_{l\mathbf{k}_0}(\mathbf{r})\cdot 2\frac{\partial}{\partial x}\psi_{j\mathbf{k}_0}(\mathbf{r})\,d\mathbf{r}$$

$$p_{ljy} = i\frac{(2\pi)^2}{\Omega}\int \psi^*_{l\mathbf{k}_0}(\mathbf{r})\cdot 2\frac{\partial}{\partial y}\psi_{j\mathbf{k}_0}(\mathbf{r})\,d\mathbf{r}$$

$$q_{lj} = i\frac{(2\pi)^2}{\Omega}\int \psi^*_{l\mathbf{k}_0}(\mathbf{r})\,\psi_{j\mathbf{k}_0}(\mathbf{r})\,d\mathbf{r}$$

The operator $\frac{\partial}{\partial x}$ behaves like B_1 while $\frac{\partial}{\partial y}$ behaves like B_2 in C_{2V} group. Combining this fact with the direct product table for C_{2v} group, we can find the matrix elements obtained in the effective Hamiltonian are the only way to make all become A_1, the full symmetric representation. This indicates that such style of effective Hamiltonian is symmetry decided.

From the 4×4 effective Hamiltonian the eigen spectrum and the weight of the four states that make up the bands can be obtained simultaneously. Since the Dirac points are formed by the linear crossing of the fourth and fifth bands, far away from the third and sixth bands in frequency, the fraction of A_1 and B_1 representation, which can be obtained for each state with given (k_x, k_y), would dominate. In this way its evolution, as is seen in Fig. 2, can be calculated.

III. Band dispersion along XM and ΓX

Along XM and ΓX, the effective Hamiltonian matrix is block diagonalized. The eigen frequency dispersion can be solved analytically. For κ_x=0 and along κ_y, we have

$$\frac{\tilde{\omega}^2_{B_1}}{c^2} = \frac{1}{2}\left[\left(\frac{\omega^2_{B_1}+\omega^2_{A_2}}{c^2}+q_{B_1B_1}\kappa_y^2+q_{A_2A_2}\kappa_y^2\right)-\sqrt{\left(\frac{\omega^2_{A_2}-\omega^2_{B_1}}{c^2}+q_{B_1B_1}\kappa_y^2-q_{A_2A_2}\kappa_y^2\right)^2+4p^2_{A_2B_1}\kappa_y^2}\right]$$

and

$$\frac{\tilde{\omega}^2_{A_1}}{c^2} = \frac{1}{2}\left[\left(\frac{\omega^2_{B_2}+\omega^2_{A_1}}{c^2}+q_{B_2B_2}\kappa_y^2+q_{A_1A_1}\kappa_y^2\right)+\sqrt{\left(\frac{\omega^2_{A_1}-\omega^2_{B_2}}{c^2}+q_{B_2B_2}\kappa_y^2-q_{A_1A_1}\kappa_y^2\right)^2+4p^2_{A_1B_2}\kappa_y^2}\right].$$

Since the band order of {B_1, A_2} and {A_1, B_2} is fixed, these two bands would inevitably cross once ω_{A_1} becomes lower than ω_{B_1}. For κ_y=0 and along κ_x, we have

$$\frac{\omega_{\pm}^2}{c^2}=\frac{1}{2}\left[\left(\frac{\omega_{A_1}^2+\omega_{B_1}^2}{c^2}+q_{B_1B_1}\kappa_x^2+q_{A_1A_1}\kappa_x^2\right)\pm\sqrt{\left(\frac{\omega_{B_1}^2-\omega_{A_1}^2}{c^2}+q_{B_1B_1}\kappa_x^2-q_{A_1A_1}\kappa_x^2\right)^2+4p_{A_1B_1}^2\kappa_x^2}\right].$$

It is easy to see that $\tilde{\omega}_{A_1}=\omega_+$ if $\omega_{A_1}>\omega_{B_1}$ and $\tilde{\omega}_{B_1}=\omega_+$ if $\omega_{B_1}>\omega_{A_1}$, meaning that band inversion occurs in this direction once the two bands touch.

IV. Effective two-band model

Since A_2 and B_2 bands are far from the degeneracy frequency, we take a procedure to reduce the effective Hamiltonian to a two-band model. Here, we drop A_2 and B_2 bands off from the effective Hamiltonian, but correct the two-band model through virtual process in which electromagnetic mode jumps from A_1 or B_1 band to A_2 or B_2 band, then back to the original band [S3, S4]. Thus the eigen problem can be written as

$$H_{11}\psi_{B_2}+H_{12}\psi_{A_1}+H_{14}\psi_{A_2}=\frac{\omega_0^2}{c^2}\psi_{B_2} \quad (1)$$

$$H_{21}\psi_{B_2}+H_{22}\psi_{A_1}+H_{23}\psi_{B_1}=\frac{\omega^2}{c^2}\psi_{A_1} \quad (2)$$

$$H_{32}\psi_{A_1}+H_{33}\psi_{B_1}+H_{34}\psi_{A_2}=\frac{\omega^2}{c^2}\psi_{B_1} \quad (3)$$

$$H_{41}\psi_{B_2}+H_{43}\psi_{B_1}+H_{44}\psi_{A_2}=\frac{\omega_0^2}{c^2}\psi_{A_2} \quad (4)$$

Here H_{ij} is the element of original four by four effective Hamiltonian matrix, and ω_0 is the degenerated frequency, which can be obtained through the equation $\tilde{\omega}_{A_1}=\tilde{\omega}_{B_1}$.

Thus by expressing ψ_{A_2}, ψ_{B_2} in terms of ψ_{A_1}, ψ_{B_1}, and substituting them back into Eq. (2) and (3), the two-band Hamiltonian matrix is determined as

$$\tilde{H}=\begin{pmatrix}H_{22} & H_{23}\\ H_{32} & H_{33}\end{pmatrix}+\frac{1}{\left(H_{11}-\frac{\omega_0^2}{c^2}\right)\left(H_{44}-\frac{\omega_0^2}{c^2}\right)-H_{41}H_{14}}\begin{pmatrix}-H_{21}\left(H_{44}-\frac{\omega_0^2}{c^2}\right)H_{21} & H_{21}H_{14}H_{43}\\ H_{12}H_{41}H_{34} & -H_{34}\left(H_{11}-\frac{\omega_0^2}{c^2}\right)H_{43}\end{pmatrix}$$

Checking the matrix element we can confirm that the diagonal terms are real and off-diagonal terms are imaginary, and all terms are functions of (κ_x, κ_y). Therefore, setting

$$\tilde{H}=\begin{pmatrix}a(\kappa_x,\kappa_y) & -ib(\kappa_x,\kappa_y)\\ ib(\kappa_x,\kappa_y) & c(\kappa_x,\kappa_y)\end{pmatrix}$$

we can expand the two-band model in the basis of Pauli matrx as follows

$$\tilde{H} = \frac{1}{2}\left(a\left(\kappa_x,\kappa_y\right)+c\left(\kappa_x,\kappa_y\right)\right)\sigma_0 + b\left(\kappa_x,\kappa_y\right)\sigma_y + \frac{1}{2}\left[a\left(\kappa_x,\kappa_y\right)-c\left(\kappa_x,\kappa_y\right)\right]\sigma_z .$$

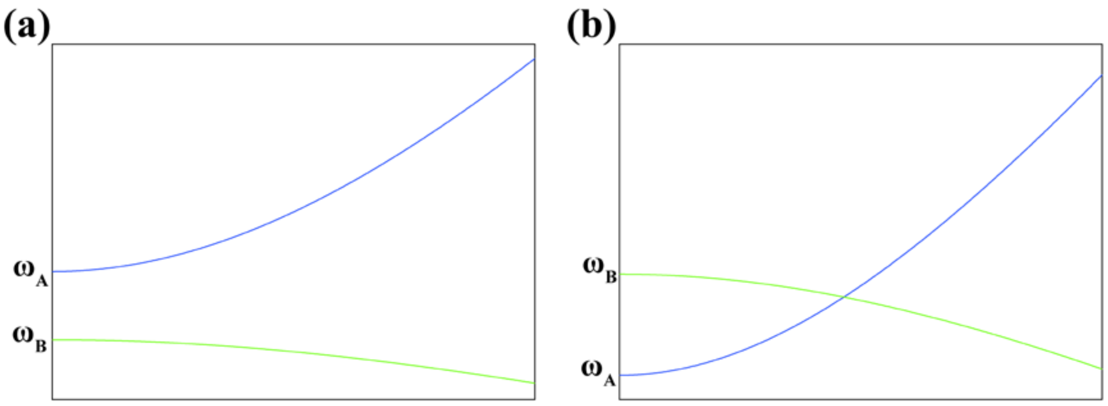

FIG. S1 The schematic diagram of two bands with different parity along a mirror reflection invariant line. In (a) a gap exists between A type and B type bands, while in (b) the two bands are tuned to have an unavoidable crossing protected by mirror symmetry.

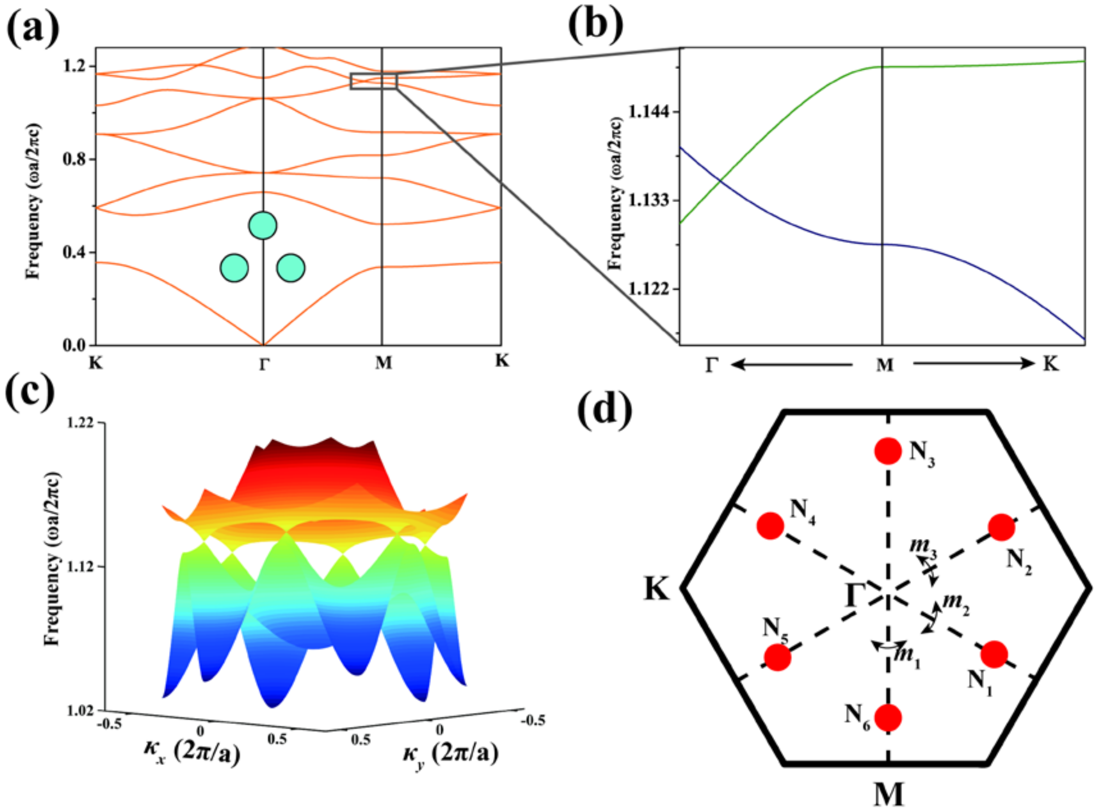

FIG. S2 Band structure of a 2D photonic crystal with a triangular lattice of dielectric cylinders with relative permittivity ε=7.6 and radius r=0.18a embedded in air. Here a is the lattice constant. (a) The band structure along high symmetry lines. Gapless topological nodal points with linear dispersions emerge along ΓM. (b) Enlarged view near the nodal point. (c) Three dimensional dispersion surface containing four topological nodal points in the first Brillouin zone. (d) The red dots mark the positions of topological nodal points {N_1, N_2, N_3, N_4, N_5, N_6} in the first Brillouin zone. The three dot lines represent the mirror operation m_1, m_2 and m_3.

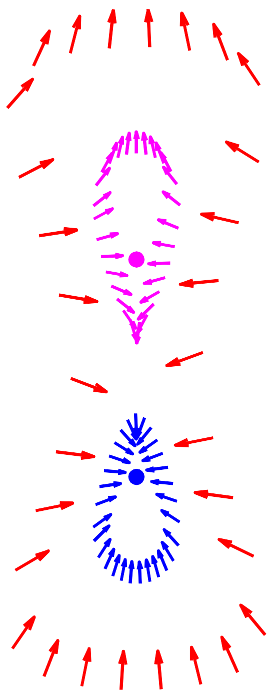

FIG. S3 (a) Topological nodal points characterized by topological vortex. The winding number of time reversal related topological vortices is opposite to each other as shown.

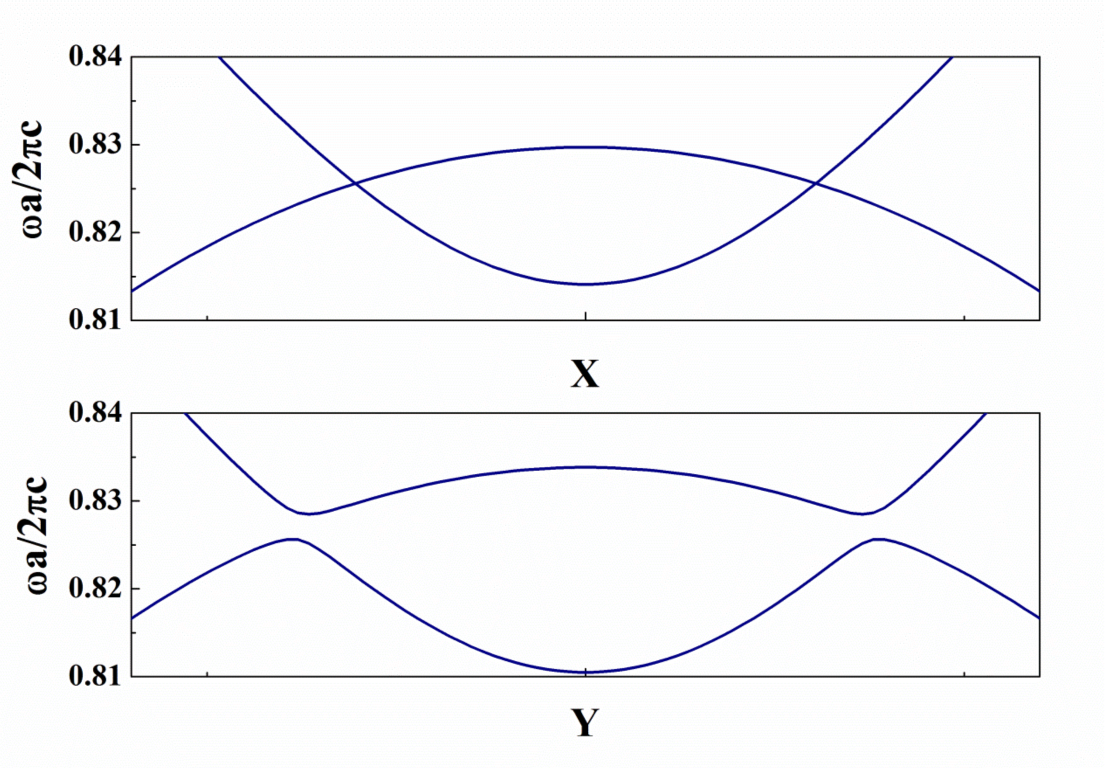

FIG. S4 The Dirac points hold when breaking all other symmetries (including time reversal symmetry) other than mirror symmetry. The magnetic field is applied along m_x mirror symmetry axis to break the time reversal symmetry. In the upper panel the Dirac points are protected by m_x while in the lower panel the Dirac points are gapped. The corresponding permeability tensor is $\mu = \begin{pmatrix} 1 & & -0.2i \\ & 1 & \\ 0.2i & & 1 \end{pmatrix}$.

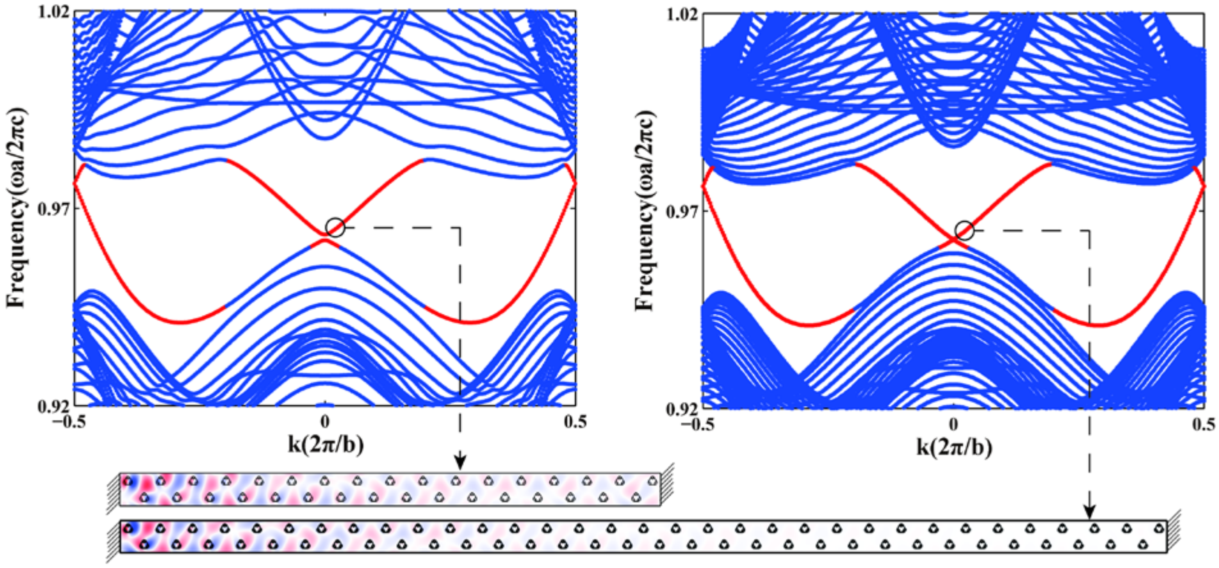

FIG. S5 The finite size effect of the edge modes. The minigap due to the coupling of edge modes induced by finite size effect in edge modes spectrum is suppressed dramatically by widening the photonic crystal ribbon, consistent with the difference between edge spectrum in right panel and left panel. The corresponding electric field distribution of these edge modes are shown at the bottom.

[S2] J. Mei, Y. Wu, C. T. Chan, and Z. Q. Zhang, Phys. Rev. B **86**, 035141 (2012).
[S3] K. Sun, W. V. Liu, A. Hemmerich, and S. D. Sarma, Nature Phys. 8, 67 (2011).
[S4] R. de Gail, M. O. Goerbig, F. Guinea, G. Montambaux, and A. H. Castro Neto, Phys. Rev. B **84**, 045436 (2011).
